# Inventory of the benthic eukaryotic diversity in the oldest European lake

**DOI:** 10.1002/ece3.7907

**Published:** 2021-07-30

**Authors:** Benjamin Wilden, Walter Traunspurger, Stefan Geisen

**Affiliations:** ^1^ Department of Animal Ecology Bielefeld University Bielefeld Germany; ^2^ Laboratory of Nematology Wageningen University Wageningen The Netherlands; ^3^ Netherlands Department of Terrestrial Ecology Netherlands Institute for Ecology (NIOO‐KNAW) Wageningen The Netherlands

**Keywords:** ancient lake, benthic eukaryotes, eDNA, functional diversity

## Abstract

We have profound knowledge on biodiversity on Earth including plants and animals. In the recent decade, we have also increased our understanding on microorganisms in different hosts and the environment. However, biodiversity is not equally well studied among different biodiversity groups and Earth's systems with eukaryotes in freshwater sediments being among the least known. In this study, we used high‐throughput sequencing of the 18S rRNA gene to investigate the entire diversity of benthic eukaryotes in three distinct habitats (littoral sediment and hard substrate, profundal sediment) of Lake Ohrid, the oldest European lake. Eukaryotic sequences were dominated by annelid and arthropod animals (54% of all eukaryotic reads) and protists (Ochrophyta and Ciliophora; together 40% of all reads). Eukaryotic diversity was 15% higher in the deep profundal than on either near‐surface hard substrates or littoral sediments. The three habitats differed in their taxonomic and functional community composition. Specifically, heterotrophic organisms accounted for 92% of the reads in the profundal, whereas phototrophs accounted for 43% on the littoral hard substrate. The profundal community was the most homogeneous, and its network was the most complex, suggesting its highest stability among the sampled habitats.

## Graphical abstract

In this study, we provide the first molecular inventory of the biodiversity in different benthic freshwater habitats of ancient Lake Ohrid. We show that eukaryotic communities, dominated by animals and protists, are fundamentally different both at a taxonomic and at a functional level between near‐surface littoral and 200 m deep profundal samples.

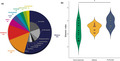

## 1 INTRODUCTION

While the biodiversity of higher plants and animals has long been appreciated (Larsen et al., 2017), the importance of the biodiversity of microorganisms in different hosts and in the environment has been recognized relatively recently (Thompson et al., 2017). Biodiversity is essential for ecosystem functioning (Loreau et al., 2001), but its decline due to anthropogenic impacts has made its documentation across the Earth's living systems all the more urgent (Johnson et al., 2017; Mihoub et al., 2017; Veresoglou et al., 2015). For microeukaryotes, particularly those in freshwater habitats, their biodiversity has yet to be examined in detail (Cazzolla Gatti, 2016; Leray & Knowlton, 2016; Xiong et al., 2021). With molecular tools, we now have the tools in our hands to map this diversity (Bik et al., 2012; Creer et al., 2016; Leray & Knowlton, 2016).

Freshwater covers only 0.8% of the land surface and accounts for only 0.01% of all water on Earth, but it harbors ~6% of all described species (Dudgeon et al., 2006; Gleick, 1996). Humans rely on freshwater sources for drinking water and irrigation as well as economic activities, especially fisheries, aquaculture, and tourism. Accordingly, many key contributors to freshwater systems have been well studied, including plankton, macrofauna, and fish, but bottom dwellers, especially small benthic organisms, have been largely neglected (Cazzolla Gatti, 2016; Kurashov, 2002). Benthic organisms can be classified as residing in one of two distinct zones that differ in their physical structure: the littoral and the profundal. The surface‐near littoral zone is a highly dynamic habitat, characterized by constant temporal variations in its physical and biological parameters (Carmignani & Roy, 2017; Galatowitsch & McIntosh, 2016). Its relatively high availability of nutrients originated from surrounding terrestrial resources such as litter and the activity of phototrophic organisms supports the establishment of a complex food chain (Brett et al., 2017). Littoral sediment is often patchy, differing with respect to grain size, macrophyte coverage, and amounts of organic matter (Lampert & Sommer, 2007). In addition, within the littoral, hard substrates such as the surfaces of stones are highly exposed to environmental variations and covered by periphyton. It mainly comprises algae but also heterotrophic components, including bacteria, fungi, protists, and small metazoans, as well as dead organic material (Weitere et al., 2018; Wetzel, 2001). By contrast, the profundal zone, defined as the area receiving <1% of photosynthetically active radiation, is a relatively stable habitat, with a constant temperature and homogeneous sediment composition (Martens, 1994; Rundle et al., 2002). These differences in the stability and complexity of the habitats usually translate to the establishment of specific communities (Mougi & Kondoh, 2012; Wilden et al., 2020).

Ancient lakes, defined as those that have existed continuously for over a million years, provide unique systems to study speciation and diversity (Brooks, 1950; Cristescu et al., 2010; Martens, 1997). In fact, ancient lakes have been identified as biodiversity hotspots (Martens, 1997; Rossiter, 2000), although the focus has so far been on single taxonomic groups of large organisms, such as fish and mollusks, except for a few model taxa such as diatoms (Albrecht & Wilke, 2008; Cvetkoska et al., 2018). In Europe, the only known ancient lake is Lake Ohrid, located on the border of Albania and North Macedonia. While its volume is much smaller (58.6 km^3^) than that of Lake Baikal and the lakes of the African rift valley (maximum volumes of >20,000 km^3^; Rossiter, 2000), Lake Ohrid is among the most species‐rich based on its total surface area, as it harbors groups of organisms with a high degree of endemism, including amphipods (90%), gastropods (78%), and ostracods (63%) (Albrecht & Wilke, 2008).

In this study, we used high‐throughput sequencing of the hypervariable V4 region of the 18S rRNA gene to profile and compare the taxonomic and functional diversity and community composition of minute eukaryotes in the littoral (sediment and hard substrates) and profundal (sediment) of Lake Ohrid. We predicted a higher taxonomic and functional diversity of eukaryotes in the two littoral habitats than in the deep profundal. At the functional level, we expected that phototrophic organisms would dominate the littoral, both its sediment and its hard substrates, whereas heterotrophic organisms would be dominant in the profundal, thus resulting in habitat‐specific communities. In addition, we hypothesized that profundal sediments host a stable community characterized by a higher network complexity, in contrast to littoral networks, which are subject to constant fluctuations and will thus be less connected.

## 2 METHODS

### 2.1 Study area

Lake Ohrid (N41°02′19″, E20°44′13″) is the oldest lake in Europe and one of the oldest lakes in the world. Formed tectonically between 1.9 and 1.3 million years ago, it is situated in the Ohrid valley, at an altitude of 693 m a.s.l., between the south‐western part of the Republic of North Macedonia and eastern Albania. It has a surface area of 358.2 km^2^, a maximum depth of 288.7 m, a water volume of 58.6 km^3^, and a shoreline length of 87.53 km (Wagner et al., 2017). The main characteristic of Lake Ohrid's ecosystem is the scarcity of nutrients, which accounts for the low level of primary production in the lake. However, due to its age, geographic isolation, and its stable ecological conditions the lake hosts a very rich biodiversity, especially its relict and endemic species (Ganoulis et al., 2000).

The sampling sites in this study were located along Lake Ohrid's northern shoreline for littoral and periphyton samples, and along a transect within a flat‐bottom section of the lake for the profundal samples (see Wilden et al., 2020 for a detailed map of sampling points).

### 2.2 Sampling procedure

All samples were obtained in April 2018. A cylindrical, modified/(diameter 6 cm; length 60 cm; Uwitec, Austria) was used to sample the sediments of Lake Ohrid at depths of 1.8–2.2 m (littoral) and 190–210 m (profundal). The distances and depths were tracked by sonar and GPS. Ten sampling sites from the littoral and ten from the profundal were sampled. From each sediment sample, the upper 5 cm of the sediment column was immediately preserved in 90% ethanol.

Also in the littoral, the periphyton from the ten sampling sites of littoral was sampled using a brush sampler to obtain quintuplicate samples at a water depth of 50 cm (Peters et al., 2005). The syringe‐like sampler scrapes off a defined area (3.14 cm^2^) on hard substrates and collects all sampled epilithic material, including biofilm‐dwelling meiofauna, without loss and without contamination by planktonic or resuspended benthic organisms and material. Five replicate samples were taken from a single stone (one stone per site). Each sample was immediately sieved (10‐µm mesh) and preserved as described for the sediment samples.

### 2.3 DNA extraction and sequencing

DNA was extracted from 10 g (total wet weight) of sediment or the total volume of periphyton that was sampled over an area of 15.7 cm^2^, using the DNeasy^®^ Power max^®^ soil kit (Qiagen N.V.) according to the manufacturer's instructions. The concentration and purity of the DNA were measured using a NanoDrop ND‐2000 spectrophotometer (NanoDrop Technologies).

The DNA was sent to Génome Québec (Montréal, Canada) for paired‐end 300 bp MiSeq sequencing. Broadly targeted primer sets were used to target eukaryotes (fungi, protists, and animals). Eukaryotic V4 regions of the 18S rRNA gene were amplified using the primer pair 616F (5′‐TTAAARVGYTCGTAGTYG‐3′) and 1132R (5′‐CCGTCAATTHCTTYAART ‐3′) (Hugerth et al., 2014).

### 2.4 Bioinformatics

The obtained raw 18S rDNA sequence reads were curated in the Hydra pipeline (de Hollander, 2017) implemented in Snakemake (Köster & Rahmann, 2012); in short, after contaminants had been filtered out and the barcodes removed, the forward reads were used for annotation. Thereafter, vsearch (Rognes et al., 2016) was used to cluster all reads into operational taxonomic units (OTUs) using the UPARSE strategy by dereplication, followed by sequence sorting by abundance (removal of singletons) and clustering using the UCLUST smallmem algorithm (Edgar, 2010). Chimeric sequences were removed using UCHIME (Edgar et al., 2011), as implemented in vsearch. To create an OTU table, all reads were mapped to OTUs using the usearch_global method (vsearch). Sequences were aligned using the SILVA database (Quast et al., 2013). Reference sequences were first trimmed of their forward and reverse primers using cutadapt (Martin, 2011). Prior to further analyses, samples with <1,000 reads were removed, and the read numbers were then recalculated as the relative abundances of the OTUs. Assignment of the OTUs to a group (phototrophic, heterotrophic, and parasitic protistans, metazoans, Streptophyta, and fungi; see the grouping and function in Appendix S1) was based on literature reports. All dinoflagellates were regarded as mixotrophic and therefore as 50% phototrophic and 50% heterotrophic. All raw sequences data were deposited in (to be submitted upon acceptance of the manuscript). In the following, all taxa are referred to in accordance with SILVA.

### 2.5 Statistical analyses

Statistical analyses performed using R 3.5.3. Visualization of the results was based on the *base* or *ggplot2* package if not stated otherwise (Wickham, 2009). The *vegan* package was used to calculate the Shannon indices for each of the three habitat types (Oksanen et al., 2018); differences in these indices were assessed using the Mann–Whitney U test. The heatmap in Figure 2 was created using *gplot* and shows the differences between groups and habitats. Every OTU was assigned to one of the following functional groups: heterotrophic, phototrophic, or parasitic. Only a few mixotrophic protists had to be counted as half heterotrophic and half phototrophic. The significance levels were assessed using Dunn's test, from the *dunn.test* package (Dinno, 2017). Habitat specificity was evaluated nonmetric multidimensional scaling (NMDS) and a similarity analysis, the latter from the *vegan* package. The role of single species in the separation of communities was analyzed using the indicator analysis from the *indicspecies* package (De Cáceres & Legendre, 2009).

In addition, co‐occurrence network analyses were performed using the packages *vegan*, *Hmisc* (Harrell & Dupont, 2020), and *igraph* (Csardi & Nepusz, 2006) to assess the connectedness of the communities from the three habitat types of Lake Ohrid. The analyses were conducted on the family level to allow the removal of artifacts arising from the inaccurate annotation of lower taxonomic levels and the discrepancy in OTUs, such that some OTUs consist of multiple species and species, in some cases, were attributed to multiple OTUs (Faust & Raes, 2012). Therefore, a family‐level correlation matrix was constructed based on Spearman's coefficient. The obtained P‐values were corrected for multiple testing using the method of Benjamini–Hochberg. The network was generated using only correlations with *ρ* > 0.7 and *p* < .05; for visualization, the Kamada and Kawai algorithm within *igraph* was used. The nodes in the reconstructed networks represent the taxonomic groups at the family level, whereas the edges (that is, connections) correspond to a strong and significant correlation between nodes. All significance thresholds were set to *α* = 0.05 and corrected using the Bonferroni–Holm method in case of multiple data usage, if not stated otherwise. The results are presented as percentages, absolute numbers, or as the mean ± standard deviation.

## 3 RESULTS

### 3.1 Diversity

**FIGURE 1:**
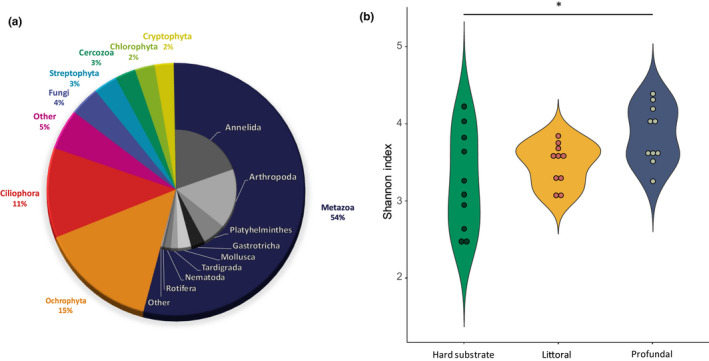
Eukaryotic community composition based on 18S rRNA gene sequencing. (a) General abundance of sequences affiliated with eukaryotic groups across all habitat types. (b) Alpha diversity of littoral hard substrates and littoral and profundal sediments displayed

Eukaryotic community profiling using 18S rRNA gene sequencing generated 2,023,727 sequences after quality filtering for downstream analysis. The sequences were assigned to 1,291 OTUs. Most of the OTUs were representative of animals (54%), dominated by Annelida (36%) and Arthropoda (30%), followed by protists (40%), mainly Ochrophyta (33%) and Ciliophora (24%) (Figure 1a). Much smaller percentages of OTUs represented fungi (4%) and plants (3%) (Figure 1a).

The alpha diversity was 15% lower on littoral hard substrates (Dunn's test, *p* = .01) than within the profundal sediment (Figure 1b). The Shannon index of the littoral hard substrates was the most variable (*SD*: 0.64) and that of the littoral sediment the least variable (*SD*: 0.28). A rarefaction analysis indicated that the estimated eukaryotic OTU richness at 200,000 sequences was ~900 for the hard substrates and ~1,050 for the littoral and profundal sediments (see rarefactions in the Figures S1–S7).

### 3.2 Abundance

**FIGURE 2:**
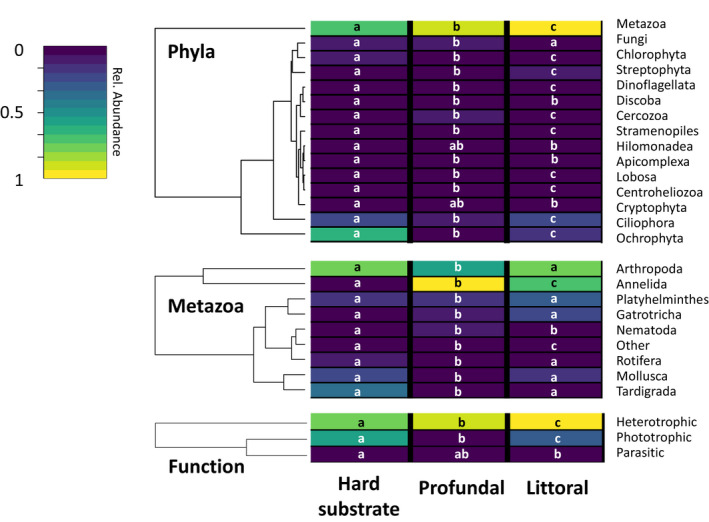
Heatmaps showing the abundances of eukaryotic phyla (a), metazoa (b), and functional categories (c) on littoral hard substrates and in the sediments of the littoral and profundal. The color key relates the heatmap colors to the relative abundance of each group. Letters indicate significantly different group abundances (Dunn's test, *p* < .05)

The abundances of metazoans, Ciliophora, and Streptophyta were highest in the littoral sediment (Dunn's test, *p* < .05; see the Appendix S2). Ochrophyta and Chlorophyta abundance decreased with depth (Dunn's test, *p* < .01). Cercozoans were most abundant in the profundal and least abundant in the littoral (Dunn's test, *p* < .05). The other phyla contributed <5% to any specific habitat (Figure 2).

Pairwise comparisons within the metazoans using Dunn's test showed that the relative abundances of mollusks, arthropods, and tardigrades were lowest in the profundal sediment (*p* < .05; see the Appendix S2), without a significant difference between the littoral habitats. Tardigrades on the littoral hard substrates were 50 times more abundant than in the profundal sediment and 12 times more abundant than in the littoral sediment (*p* < .05). Arthropods were roughly 30% less abundant in profundal than in littoral habitats (*p* < .05). Compared to the profundal sediment, mollusks were 2.3‐fold as abundant in the littoral sediment and 3.8‐fold more abundant on littoral hard substrates (*p* < .05). Annelids were 20 times less abundant on the hard substrates (*p* < .01) than within sediment habitats. Gastrotrichs were twice as abundant in the littoral sediment than in the profundal sediment or on littoral hard substrates (*p* < .05).

### 3.3 Functions

Pairwise comparisons of the relative abundances of the functional groups indicated a decreasing abundance of phototrophic organisms with depth (Figure 2; Dunn's test, *p* < .05; also see the Appendix S2). The abundance peak on the hard substrates was 2.2‐fold that of the littoral sediment and 9‐fold that of the profundal sediment. Heterotrophic organisms were the most dominant in the profundal sediment followed by the littoral sediment and littoral hard substrates (Dunn's test, *p* < .05) but the absolute differences were <25%. Parasites differed significantly only between the littoral habitats (Dunn's test, *p* < .05), with a threefold higher abundance on the hard substrates.

### 3.4 Community composition

**FIGURE 3:**
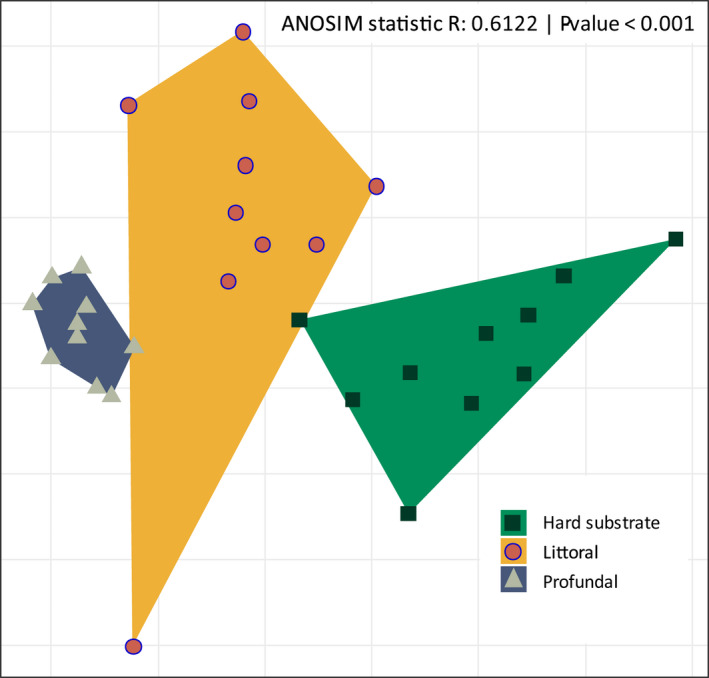
Nonmetric multidimensional scaling (NMDS) plot based on the OTUs. The Bray–Curtis similarity was calculated using the read abundances from the profundal and littoral sediments and from littoral hard substrates (stones). Shapes and colors represent the three sampling sites: littoral hard substrates (green) and the sediments of the littoral (orange) and profundal (blue). Each habitat type cluster is also indicated by polygons

The three habitats significantly differed in their community composition (ANOSIM, *p* < .001), leading to separate clusters along the gradient (Figure 3). Indicator analyses revealed that Cyrtophoria (*p* = .001), Ulvophyceae (*p* = .002), and Microthamniales (*p* = .041) were indicative of hard substrates. Among the members of these taxa, >95% were found only on the hard substrates. Although Prostomatea occurred in only half of the littoral sediment samples, this group was an indicator for this habitat (*p* = .031), as 81.2% of all Prostomatea assigned reads were obtained from the littoral sediment. Most indicator taxa were from the profundal, with many occurring (almost) entirely in that habitat. Gregarines (*p* = .001) and Euglyphida (*p* = .001) were present in every profundal sample. Planomonadidae (*p* = .014), Aphragmophora (*p* = .005), Diplonemea (*p* = .001), Nolandida (*p* = .007), and Limnofilida (*p* = .010) were detected in at least 90% of the profundal samples, Colpodellidae (*p* = .009) and Spumellarida (*p* = .006) in ~70%, and Marimonadida (*p* = .024), Arcellinida (*p* = .009), and Dactylopodida (*p* = .030) in >50%.

### 3.5 Network

**FIGURE 4:**
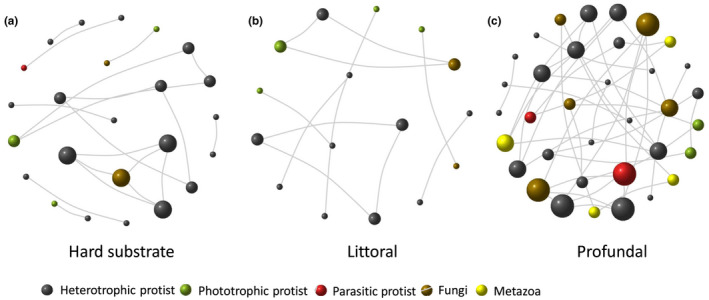
Network co‐occurrence analysis of all eukaryotes from littoral hard substrates and in the littoral and profundal sediments. A connection was defined as Pearson's correlation >0.7 (correlation: black edges) and statistically significant (*p* < .05). Each node represents a different eukaryotic family, and the size of the node is proportional to the number

Network complexity (here defined by the average changes in network properties, especially nodes, edges, and community hubs; Table 1) was highest in the profundal sediment (35 nodes; 39 edges; 12 communities; Figure 4b; Table 1) and lowest in the littoral sediment (16 nodes; 11 edges; seven community hubs; Figure 4c), followed by the littoral hard substrates (24 nodes; 19 edges; 10 communities; Figure 4a).

**TABLE 1:** Correlations and topological properties of the networks

Network properties	Hard substrate	Profundal	Littoral
Number of nodes^a^	24	35	16
Number of edges^b^	19	39	11
Modularity^c^	0.83	0.86	0.81
Number of communities^d^	10	12	7
Network diameter^e^	1	1	1
Average path length^f^	1	1	1
Degree^g^	38	78	22

^a^ Taxon (at family level) with at least one significant (*p* < .05) and strong (*ρ* > 0.7) correlation.

^b^ Number of connections/Spearman correlations.

^c^ The capability of the nodes to form highly connected communities, that is, a structure with high density of between nodes connections.

^d^ A community is defined as a group of nodes densely connected internally.

^e^ The longest distance between nodes in the network, measured in number of edges.

^f^ Average network distance between all pair of nodes or the average length of all edges in the network.

^g^ The number of connections in the network.

Four metazoan taxa were part of the network of the profundal, but they were absent from the networks of the littoral sediment and hard substrates. Parasites were not part of the littoral sediment network but were present on the hard substrates and contributed substantially to the profundal network. Two phototrophic protists also occurred in the profundal network. Nevertheless, the networks differed when reproduced at the OTU level (see the Appendix S3). On an OTU basis, the densest network was that of the littoral sediment (311 nodes; 3,140 edges) followed by the hard substrates (252 nodes; 557 edges) and the profundal network (176 nodes; 348 edges).

## 4 DISCUSSION

Our integrative study of the taxonomic and functional diversity of small eukaryotes in the oldest lake in Europe revealed distinct communities in littoral and profundal habitats that are more diverse and likely stable in the profundal.

The higher diversity of eukaryotes in the deep profundal sediment than in the littoral habitats contradicted with our main hypothesis but also the long‐standing literature on Lake Ohrid (Albrecht & Wilke, 2008; Stanković, 1960). That result also differed from a meiofauna‐based study of the exact same samples, which found the profundal to be less diverse than the littoral (Wilden et al., 2020). Differences between our sequence‐based analysis and previous morphologically based surveys can be attributed to methodological biases inherent to both approaches, especially when comparing “abundance” of multicellular organisms. For example, different markers will likely reveal slightly different community compositions due to PCR biases but the overall patterns are likely to remain (Schenk et al., 2020). Some biases such as sampling bias, taphonomic processes, potential mixing of substrates, sedimentation of actual biomass from the water column occur equally in morphological and molecular approaches. The sequence‐based analyses performed here provide relative abundance data that correlate more with biomass than total abundance data (Schenk et al., 2019, 2020). However, our molecular analysis on directly extracted DNA did not focus on animals as previous studies, but also included protists and other hardly extractable organisms (Geisen & Bonkowski, 2018). Nevertheless, the total amount of observed OTUs was lower than otherwise reported for freshwater habitats (Debroas et al., 2017). Other patterns found here are confirmed by previous studies. For example, oligochaetes dominated the OTU numbers and relative abundance of all eukaryotes, confirming the idea that Lake Ohrid is an oligochaete lake (Stanković, 1960).

Our hypothesis of a high proportion of phototrophic organisms in the littoral sediment and on the hard substrates was supported, but even in the lightless profundal ~7% of the read abundance represented phototrophic organisms. The latter can be explained by sedimentation of the resting stages and nondegraded eDNA originating from littoral organisms (Pawlowski et al., 2011; Piredda et al., 2017). The highest relative abundance of heterotrophs in the profundal was expected and found to account for 92% of all profundal reads. Nevertheless, heterotrophs also dominated with almost 80% of the littoral sediment reads. This may have been due to the import of terrestrial carbon in the littoral, which is also supported by multiple OTUs assigned to riparian vegetation (Jones et al., 2018). The relative abundance of parasites never exceeded 1% of the reads, but parasites are known to be less abundant and less species‐rich in freshwater than in marine systems (Marcogliese, 2001).

Our results support the hypothesis that profundal sediments host a more stable community that translates into higher network complexity, whereas dynamic littoral networks are less connected. The profundal community was more homogeneous, as previously also determined for meiofauna (Wilden et al., 2020), but also more diverse than the littoral communities. The differences in the communities of the three habitats were caused by multiple OTUs or species, and in some cases entire orders, that were limited to or more strongly associated with one versus the other habitats. The profundal harbored the largest number of unique taxa and thus comprised a true profundal community consisting of species adapted to life in deep water (Stanković, 1960). In general, highly connected networks show that species are more likely to occur at the same sites within a habitat (Barberán et al., 2012). This may suggest, for instance, that communities in certain habitats that display high levels of connectivity. In a previous study, high network complexity was shown to result in a high stability (Mougi & Kondoh, 2012). Multiple types of interactions are another indicator of stability (Mougi & Kondoh, 2012), but in this study we used co‐occurrence networks that were not necessarily display direct interactions. The littoral sediment was the least complex at the family level but the most complex at the OTU level. This unusual finding might be the result of Lake Ohrid's unique endemic richness, as multiple members of a few families may have radiated within the lake and were thus possibly underrepresented in the family‐based analysis (Albrecht & Wilke, 2008). Especially for Lake Ohrid, this offers opportunities for future investigations as its evolutionary history is very well documented (Wagner et al., 2019; Wilke et al., 2020).

In summary, we show an unexpectedly lower taxonomic and functional diversity of eukaryotes in the two littoral habitats than in the deep profundal. At the functional level, we found habitat‐specific communities, but heterotrophic instead of phototrophic organisms dominated the littoral habitats, both its sediment and its hard substrates. In addition, we found evidence that profundal sediments host a stable community characterized by a higher network complexity, in contrast to littoral networks. Nevertheless, this first inventory of eukaryotes of Lake Ohrid revealed the need for more thorough investigations for a better understanding of Earth´s biodiversity.

## CONFLICT OF INTEREST

All authors declare no conflict of interest.

## AUTHOR CONTRIBUTIONS

**Benjamin Wilden:** Conceptualization (equal); Data curation (equal); Formal analysis (lead); Investigation (lead); Methodology (supporting); Project administration (equal); Resources (equal); Visualization (lead); Writing‐original draft (lead); Writing‐review & editing (equal). **Walter Traunspurger:** Conceptualization (supporting); Funding acquisition (equal); Investigation (supporting); Project administration (supporting); Supervision (supporting); Writing‐original draft (supporting); Writing‐review & editing (supporting). **Stefan Geisen:** Conceptualization (equal); Data curation (equal); Formal analysis (supporting); Investigation (supporting); Methodology (equal); Project administration (supporting); Supervision (equal); Visualization (supporting); Writing‐original draft (equal); Writing‐review & editing (supporting).

## Supporting information

Appendix S1

Appendix S2

Appendix S3

Figures S1‐S7

## Data Availability

DNA sequences are available at NCBI: PRJNA661589. Functional grouping and OTU raw data: Appendix S1 of this study.
