# Supplementary material for: Inventory of the benthic eukaryotic diversity in the oldest European lake
Source: Ecol Evol. 2021 Jul 30;11(16):11207–15. doi: 10.1002/ece3.7907 (PMC8366835; doi:10.1002/ece3.7907)
Supplement: Supplementary file 4 — Figures S1‐S7 [file ECE3-11-11207-s002.docx]

 Fig. S1: Overview of species within each sample in correspondence to the read number. The letters P represent profundal samples, L littorsal samples and H hard substrate samples. Fig. S2: Estimated species richness on profundal samples by rarefaction. Fig. S3: Estimated species richness on littoral samples by rarefaction.

 Fig. S4: Estimated species richness on littoral hard substrate samples by rarefaction.

 Fig. S5: Estimated species richness on profundal individual reads by rarefaction.

 Fig. S6: Estimated species richness on littoral sediment individual reads by rarefaction.

 Fig. S7: Estimated species richness on littoral hard substrate individual reads by rarefaction.
